# Diagnostic performance of automated plasma amyloid-β assays combined with pre-analytical immunoprecipitation

**DOI:** 10.1186/s13195-022-01071-y

**Published:** 2022-09-07

**Authors:** Hans-W. Klafki, Jonathan Vogelgsang, Ekaterina Manuilova, Chris Bauer, Alexander Jethwa, Hermann Esselmann, Anke Jahn-Brodmann, Dirk Osterloh, Ingolf Lachmann, Benedict Breitling, Carolin Rauter, Niels Hansen, Caroline Bouter, Stefan Palme, Johannes Schuchhardt, Jens Wiltfang

**Affiliations:** 1 Department of Psychiatry and Psychotherapy, University Medical Center Goettingen (UMG), Georg-August-University, Von-Siebold-Str. 5, 37075 Goettingen, Germany; 2 Current address: McLean Hospital, Department of Psychiatry, Harvard Medical School, Translational Neuroscience Laboratory, Belmont, MA 02478 USA; 3 Roche Diagnostics GmbH, 82377 Penzberg, Germany; 4 MicroDiscovery GmbH, Marienburger Strasse 1, 10405 Berlin, Germany; 5 Roboscreen GmbH, Hohmannstrasse 7, 04129 Leipzig, Germany; 6 Department of Nuclear Medicine, University Medical Center Goettingen (UMG), Georg-August-University, 37075 Goettingen, Germany; 7 German Center for Neurodegenerative Diseases (DZNE), 37075 Goettingen, Germany; 8 Neurosciences and Signaling Group, Institute of Biomedicine (iBiMED), Department of Medical Sciences, University of Aveiro, 3810-193 Aveiro, Portugal

**Keywords:** Alzheimer’s disease, Biomarker assay, Plasma Amyloid-β 42/40, Immunoprecipitation, Pre-analytical sample workup

## Abstract

**Background:**

Measurements of the amyloid-β (Aβ) 42/40 ratio in blood plasma may support the early diagnosis of Alzheimer’s disease and aid in the selection of suitable participants in clinical trials. Here, we compared the diagnostic performance of fully automated prototype plasma Aβ42/40 assays with and without pre-analytical sample workup by immunoprecipitation.

**Methods:**

A pre-selected clinical sample comprising 42 subjects with normal and 38 subjects with low cerebrospinal fluid (CSF) Aβ42/40 ratios was studied. The plasma Aβ42/40 ratios were determined with fully automated prototype Elecsys® immunoassays (Roche Diagnostics GmbH, Penzberg, Germany) by direct measurements in EDTA plasma or after pre-analytical Aβ immunoprecipitation. The diagnostic performance for the detection of abnormal CSF Aβ42/40 was analyzed by receiver operating characteristic (ROC) analysis. In an additional post hoc analysis, a biomarker-supported clinical diagnosis was used as a second endpoint.

**Results:**

Pre-analytical immunoprecipitation resulted in a significant increase in the area under the ROC curve (AUC) from 0.73 to 0.88 (*p* = 0.01547) for identifying subjects with abnormal CSF Aβ42/40. A similar improvement in the diagnostic performance by pre-analytical immunoprecipitation was also observed when a biomarker-supported clinical diagnosis was used as a second endpoint (AUC increase from 0.77 to 0.92, *p* = 0.01576).

**Conclusions:**

Our preliminary observations indicate that pre-analytical Aβ immunoprecipitation can improve the diagnostic performance of plasma Aβ assays for detecting brain amyloid pathology. The findings may aid in the further development of blood-based immunoassays for Alzheimer’s disease ultimately suitable for screening and routine use.

**Supplementary Information:**

The online version contains supplementary material available at 10.1186/s13195-022-01071-y.

## Background

Alzheimer’s disease (AD) is a progressive neurodegenerative disorder and represents the most common cause of dementia [1]. The neuropathological changes in AD brain, including extracellular neuritic plaques comprising amyloid-β (Aβ) peptides [2, 3], neurofibrillary tangles composed of abnormally phosphorylated tau [4], and selective synaptic and neuronal damage and losses [5], start to develop many years before the manifestation of clinical symptoms [6, 7]. It is clear that any potentially disease-modifying AD treatment has to start as early as possible in the course of the disease to be most effective. Affordable, reliable, and robust biomarker tests may serve as essential tools for screening, to support the early diagnosis, to identify subjects at high risk, and to aid in selection of suitable participants in clinical trials of novel drug candidates [8].

Well-documented biomarkers of cerebral amyloid pathology are increased signals on amyloid positron emission tomography (amyloid PET) (for a recent review, see e.g. [9]), low Aβ42 concentrations in cerebrospinal fluid (CSF), a reduced CSF Aβ42/Aβ40 ratio [10, 11], and increased CSF phospho-Tau/Aβ42 and total Tau/Aβ42 ratios [12–14]. However, amyloid PET is very expensive and not easily accessible, and CSF analysis requires lumbar puncture, which is not a minimally invasive procedure. Therefore, cost-effective and easily accessible blood-based biomarkers surrogating brain amyloid are highly desirable and have been searched for very actively [8]. Very recently, immunoprecipitation followed by mass spectrometry (IP-MS) for measuring the Aβ42/40 ratio in blood plasma as a surrogate biomarker of brain amyloid accumulation was successfully developed and validated [15].

For broader implementation in primary care and screening, highly standardized, automated, immunological, high-throughput plasma Aβ assays may have several advantages, provided sufficient accuracy, cost effectiveness, and reproducibility between laboratories and assay batches can be achieved. Furthermore, reliable cut points that are valid across different laboratories would be highly desirable. In a recent study, the performance of fully automated prototype Elecsys® plasma Aβ42 and Aβ40 immunoassays (Roche Diagnostics GmbH, Penzberg, Germany) was investigated in two different clinical cohorts that were dichotomized with reference to the CSF Aβ42/40 ratio. The area under the receiver operating characteristic (ROC) curve (AUC) for predicting Aβ status for a model including plasma Aβ40 and Aβ42 reached 0.80 in the discovery cohort and 0.86 in the validation cohort, respectively. The authors concluded that in the future optimized versions of blood-based Aβ assays might be useful (e.g. in pre-screening for cohort selection for clinical AD trials) [16]. In direct head-to-head comparison, the IP-MS plasma Aβ42/40 assay developed at Washington University outperformed four different Aβ42/40 immunoassays including Elecsys, a different IP-MS method developed at the University of Gothenburg and an antibody-free liquid-chromatography MS method for identifying subjects with abnormal CSF Aβ42/40. Among the four tested immunoassays, the fully automated Elecsys reached the highest AUCs in the ROC analyses [17].

In general, the measurement of Aβ peptides in blood is technically challenging because of low Aβ concentration [18, 19] and the presence of potentially interfering blood components [20]. In previous work, a two-step immunoassay was developed for measuring the plasma Aβ42/40 ratio including pre-analytical Aβ IP in the presence of three different detergents and with an antibody against the Aβ N-terminus followed by quantification of Aβ38, Aβ40, and Aβ42 with a commercially available chemiluminescence multiplex Aβ-immunoassay [21]. The pre-analytical sample workup by Aβ IP was intended to ameliorate potential matrix interferences and thus facilitate the measurement of Aβ in blood plasma. In our current study, we assessed the compatibility of the magnetic bead Aβ IP performed on a semi-automated liquid handling instrument with subsequent fully automated plasma Aβ assays on the Elecsys platform and compared the diagnostic performance of the Aβ42/40 ratio measured on Elecsys without and with pre-analytical Aβ-IP. For additional comparisons, two-step immunoassays employing the MesoScale Discovery (MSD) chemiluminescence V-PLEX Aβ panel 1 (6E10) multiplex assay for Aβ quantification in IP-eluates were performed.

## Materials and methods

### Preparation of functionalized magnetic beads and semi-automated Aβ-immunoprecipitation

Functionalized magnetic beads for Aβ-immunoprecipitation (Aβ-IP) were prepared by covalently coupling the monoclonal antibody (mAb) 1E8 (nanoTools, Teningen, Germany) with Dynabeads M-280 Sheep anti-Mouse IgG (Invitrogen/ThermoFisher Scientific, Waltham, MA, USA) according to the manufacturer’s instructions and as described in detail previously [21]. The semi-automated pre-analytical magnetic bead IP was performed on a CyBio FeliX liquid handling Instrument (Analytik Jena) equipped with BioShake 3000-Telm (Q Instruments, Germany) and ALPAQUA MAGNUM FLX Enhanced Universal Magnet Plate (Beverly, MA, USA). The current IP-protocol was a modification of a previously published version [22]. In brief, EDTA-blood plasma samples that had been stored frozen at -80°C in Matrix vials (Thermo Fisher) were thawed, mixed vigorously (5 × 10 s), and centrifuged for 10 min at 10,000 × g at room temperature in a fixed angle rotor for removal of insoluble material. 200 μL of the plasma supernatant was mixed with 200 μL of H_2_O and 100 μL of 5 × IP-buffer concentrate containing 250 mM HEPES/NaOH, pH 7.4, 750 mM NaCl, 2.5% Igepal CA630, 1.25% sodium deoxycholate; 0.25% SDS, and 1 tablet of Complete Mini Protease inhibitor cocktail (Roche Diagnostics GmbH, Mannheim, Germany) per 2 mL. After addition of 25 μL of 1E8 magnetic beads to each sample, the IP was carried out on the CyBio FeliX instrument overnight at approximately 17 °C with regular mixing. The supernatant was removed, and the beads were washed three times for 5 min with 1 mL per well of PBS containing 0.1% w/v BSA and once for 3 min with 1 mL of 10 mM Tris-HCl, pH 7.5. The bound Aβ peptides were eluted from the magnetic-bead immune complexes by adding 2 × 25 μL of 20 mM bicine, pH 7.6/0.6% CHAPS and heating for 5 min at a set temperature of 99 °C and 1100 RPM in Deep Well MegaBlock, 96 well plates, 1.2 ml PP (Sarstedt, Germany) without lid (the effective temperature was approximately 91–94°C). Per sample, approximately 38 μL (estimated range: 36–40 μL) of bead-free eluate were obtained. For the measurements on the Elecsys platform, the eluates were frozen at − 80°C and transported on dry ice. For measurements on the Aβ panel 1 (6E10) multiplex assay, the eluates were mixed with 190 μL of Diluent-35 and stored frozen at − 80°C until use, as described before [22]. With regard to the obtained volume of approx. 38 μL of IP-eluate after heating, this corresponded to 6-fold dilution.

### Quantification of Aβ40 and Aβ42 on the Elecsys platform

**Fig. 1:**
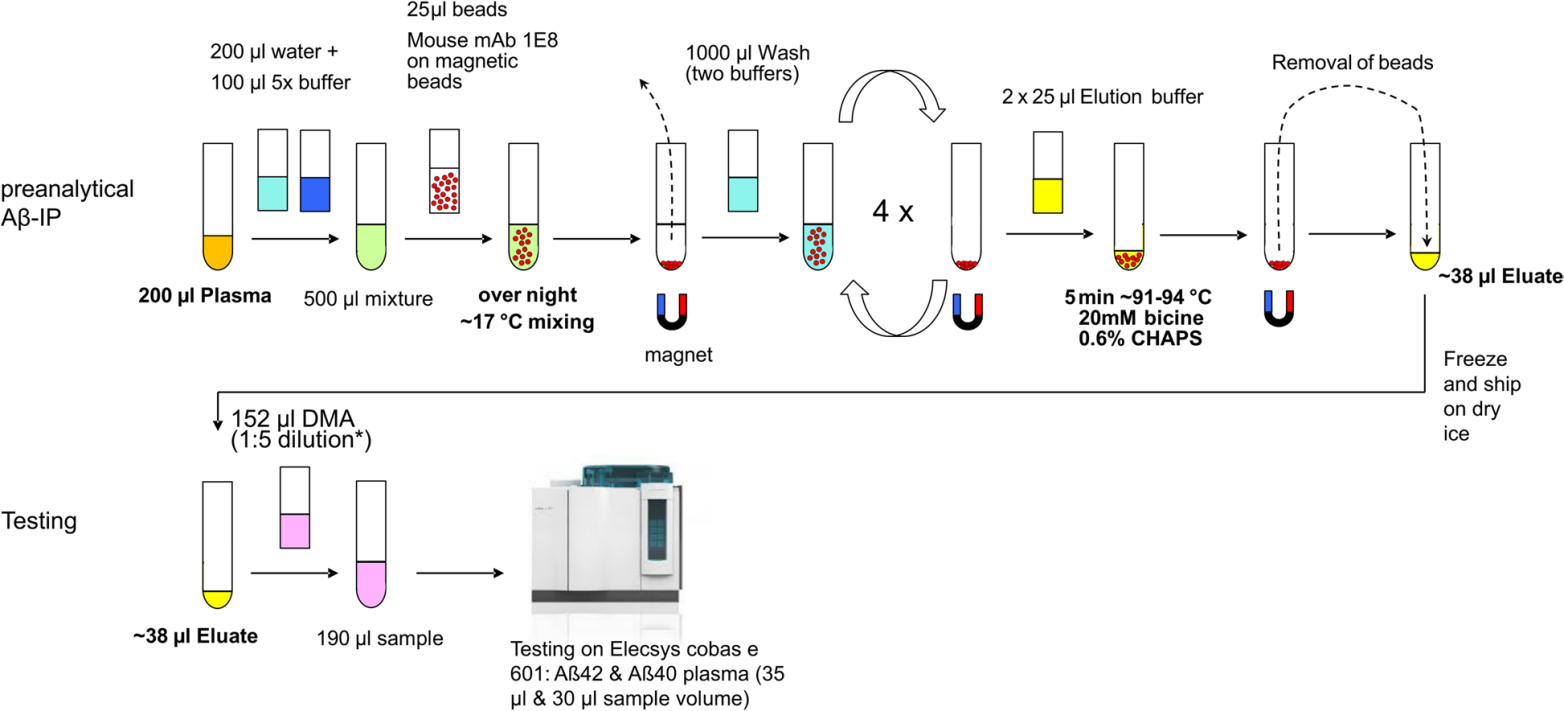
Schematic representation of the pre-analytical process before automated testing of IP-eluates from blood plasma. Aβ, amyloid-β; IP, immunoprecipitation

EDTA plasma patient samples with and without prior pre-analytical Aβ-IP were measured in single determination on the automated Elecsys cobas e 601 instrument with Aβ40 and Aβ42 prototype assays, essentially as described previously [16]. The quantitative range of the Aß40 assay is from 0.01 ng/ml to 8.9 ng/ml; the respective range of the Aß42 assay from 1.0 pg/ml to 94 pg/ml. The samples were randomized and operators were blinded for the diagnostic classification. Samples without prior IP (plasma) were placed directly on the instrument. IP-eluates were measured after manual addition of 152 μL Elecsys Diluent MultiAssay (DMA, Ref 03609987 190) to approximately 38 μL of IP-eluate. The resulting 5-fold diluted IP-eluates had adjusted final volumes of approximately 190 μL, each, which was almost identical to the input plasma volume in the IP reaction (200 μL). The entire workflow for this two-step procedure (magnetic bead IP followed by automated Aβ quantification on the Elecsys platform) is summarized in Fig. 1.

### Measurements of IP-eluates on the MSD Aβ panel 1 multiplex assay

For the Aβ measurements in IP-eluates on the MSD V-PLEX Aβ panel 1 (6E10) multiplex assay (Meso Scale Discovery, Rockville, MD, USA), IP-eluates were used that had been diluted 6-fold in Diluent-35 immediately after the heat-elution in Bicine/CHAPS and stored frozen at − 80°C until the actual measurements [22]. The Aβ multiplex assay was performed according to the manufacturer’s instructions and as described previously [21]. According to the Certificate of Analysis provided with the assay kit, the lower limit of quantification (LLOQ) of the kit lot in use was 50 pg/mL for Aβ40 and 3.13 pg/mL for Aβ42. All of the measured diluted IP-eluates produced Aβ40 and Aβ42 signals in the quantitative assay range (> LLOQ). The measured concentrations ranged from 62.9 to 235.4 pg/mL for Aβ40 and 8.4 pg/mL to 26.6 pg/mL for Aβ42.

### Study approval and study cohort

The pseudonymized collection of biological samples and clinical data and their use in biomarker studies was approved by the ethics committee of the University Medical Center Goettingen (9/2/16). All subjects or their legal representatives gave their informed consent prior to inclusion. Biomaterial sampling and data collection were conducted according to the revised Declaration of Helsinki and good clinical practice guidelines. Briefly, plasma was sampled in EDTA tubes, centrifuged for 10 min at 2000 × g and stored frozen at − 80°C until use.

### Classification of the study participants

The participants of the study were pre-selected from our local biobank and dichotomized according to their CSF Aβ42/40 ratios. The CSF concentrations of Aβ42 and Aβ40 had been determined routinely with commercial Aβ-ELISA kits in the Laboratory of Clinical Neurochemistry and Neurochemical Dementia Diagnostics, Department of Psychiatry and Psychotherapy, University of Erlangen-Nuremberg, Germany. The subjects were classified according to the clinical cut-off point into the diagnostic groups Aβ-positive (CSF Aβ42/40 ≤ 0.050) and Aβ-negative (CSF Aβ42/40 > 0.050). Both groups included cognitively unimpaired subjects, patients with mild cognitive impairment and demented patients. The characteristics of the study cohort and CSF biomarker measures are summarized in Table 1.

**Table 1:** Description of the study cohort and baseline CSF biomarker data

	All (***n*** = 80)	Aβ+^1^ (***n*** = 38)	Aβ−^1^ (***n*** = 42)	***p***-value^2^
Age [years, median ± MAD]	68 ± 8.9	71 ± 7.4	65 ± 5.9	0.0056
Female	46 (57.5%)	23 (60.53%)	23 (54.76%)	
Male	34 (42.5%)	15 (39.47%)	19 (45.24%)	
ApoE4 carrier	37 (46.25%)	29 (76.3%)	8 (19.05%)	
CSF Aβ42/40 [median ± MAD]	0.062 ± 0.031	0.036 ± 0.008	0.078 ± 0.010	< 0.0001
CSF Aβ42 [pg/mL, median ± MAD]	706.5 ± 354.3	467.5 ± 161.6	893.0 ± 276.5	< 0.0001
CSF Aβ40 [pg/mL, median ± MAD]	11820.0 ± 3724.3	12423.0 ± 4548.6	10835.5 ± 3407.8	0.0803
CSF t-Tau [pg/mL, median ± MAD]	328.0 ± 197.2	476.5 ± 172.7	205.0 ± 71.9	< 0.0001
CSF pTau-181 [pg/mL, median ± MAD]^3^	51.7 ± 23.9	74.6 ± 23.7	36.5 ± 9.9	< 0.0001

^1^The clinical sample was dichotomized according to the CSF Aβ42/Aβ40 ratio. Aβ-positive (Aβ+): CSF Aβ42/40 ≤ 0.050; Aβ-negative (Aβ−): CSF Aβ42/40 > 0.050

^2^Mann-Whitney-test *p*-values for the comparison between the groups Aβ+ and Aβ−

^3^In one case, the pTau 181 concentration was reported as <15.6 pg/mL. To be included in the statistical analysis, this value was artificially set to a fixed value of 15.6 pg/mL

*Aβ* amyloid-β, *CSF* cerebrospinal fluid, *MAD* median absolute deviation scaled with factor 1.4826, *pTau* phospho-Tau, *t-Tau* total Tau

#### Second endpoint

In additional exploratory post hoc ROC analyses we tentatively applied a different endpoint, which was intended to more closely reflect the situation in a clinical setting. To that end, and considering the recent recommendations of Dubois and colleagues [23], the study participants were classified on the basis of available clinical, psychometric and neuroimaging biomarker data (including amyloid-PET and fluorodeoxyglucose (FDG) PET, for *n* = 16 subjects and 36 subjects respectively) plus clinical observations. Details can be found in the additional file (supplementary information).

### Statistical analysis

Biomarkers were described and compared using median and MAD (median absolute deviation scaled with factor 1.4826). The latter provides a robust estimate of standard deviation. Associations between biomarker values were assessed using Spearman’s correlation coefficient and visualized using scatter plots. Systematic concentration differences between the two sample treatment groups were estimated as the following: For each sample percentage, differences were calculated as (*Y* − *X*)/*X* × 100% (*X* = concentration in samples without pre-treatment, *Y* = in samples with pre-treatment). Median percentage deviations and ranges (Min–Max) of single deviations were derived. Comparison of Elecsys vs. MSD numeric outputs was performed using Spearman’s correlation coefficients and scatter plots. The biomarker levels in the diagnostic groups Aβ-positive and Aβ-negative were compared using Mann-Whitney test. Due to the exploratory character of this study, unadjusted *p*-values are reported. The ability of biomarkers to detect amyloid positivity was evaluated using ROC-AUC analysis. 95% confidence intervals of AUC estimates were calculated using DeLong method. AUC values were compared using DeLong test. For bivariate classification of Aβ-positive vs. Aβ-negative, including the Aβ42/Aβ40 ratio and the ApoE4 allele frequency in the model, we performed logistic regression. To avoid overfitting, a leave-10-out cross-validation was applied. For all three methodological approaches, namely (i) Elecsys measurements in plasma without pre-analytical IP, (ii) Elecsys measurements in IP-eluates, and (iii) MSD measurements in IP-eluates, we calculated logistic regression single marker classifications (considering the Aβ42/Aβ40 ratio, only) and bivariate classifications including the Aβ42/Aβ40 ratio plus the ApoE4 allele frequency.

## Results

### Comparison of Elecsys plasma Aβ measurements without and with pre-analytical magnetic bead Aβ IP

**Fig. 2:**
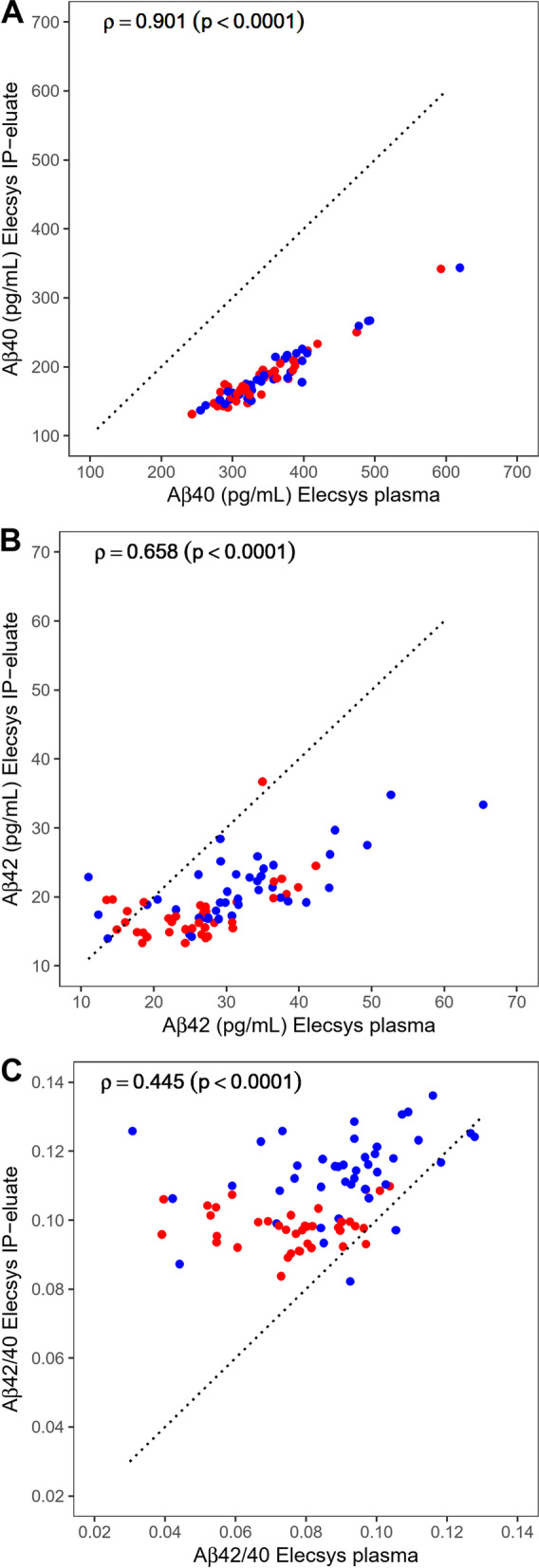
Correlations between direct plasma Aβ measurements on Elecsys and measurements after immunoprecipitation (IP). The measured concentrations of **A** Aβ40, **B** Aβ42, and **C** the Aβ42/40 ratio in diluted IP-eluates (Elecsys IP-eluate, Y) are plotted against the corresponding measurements in plasma (Elecsys plasma, X). Points are colored by diagnostic group (red = Aβ+, blue = Aβ−). The diagonal dashed lines correspond to the identity lines. Spearman correlation coefficients and *p*-values are indicated. Aβ, amyloid-β; Aβ+, Aβ-positive: CSF Aβ42/40 ≤ 0.050; Aβ−, Aβ-negative: CSF Aβ42/40 > 0.050

In total, *n* = 80 EDTA-plasma samples were analyzed on Elecsys, each one with and without pre-analytical Aβ-IP. The Aβ40 concentrations measured in plasma without pre-treatment were positively and strongly correlated to those determined in diluted IP-eluates derived from pre-analytical magnetic bead Aβ IP (Spearman’s *ρ* = 0.901, *p* < 0.0001, Fig. 2a). The correlation between the Aβ42 concentrations in plasma (direct measurements) and those in diluted IP-eluates was less pronounced (Spearman’s *ρ* = 0.658, *p* < 0.0001, Fig. 2b). Regarding the Aβ42/40 ratios, again, we observed a positive correlation between direct plasma measurements and IP-eluates (Fig. 2c). The corresponding correlation coefficient was 0.445 (Spearman’s *ρ*) and thus smaller than those of the individual Aβ peptides.

In general, the measured concentrations of Aβ40 and Aβ42 in the diluted IP-eluates were substantially lower than those in plasma without pre-treatment. Assuming quantitative recoveries over the entire workflow and taking into account the starting volume of 200 μL of plasma prior to IP and the respective final volume of 190 μL ready for testing, we expected an overall 5% increase in the measured concentrations compared to the direct measurements. However, the observed median percentage difference between Aβ40 levels in samples with and without pre-treatment was − 47% (min–max: − 55 to − 40%). The differences of Aβ42 levels were less consistent: While the median percentage difference was − 35%, the individual deviations ranged from − 53 to +108%. Overall, the entire workflow did not concentrate the two analytes compared to untreated plasma samples. Due to the different size of the pre-treatment effects for Aβ40 and Aβ42, the resulting numerical Aβ42/40 ratios were higher in the pre-treated samples.

### Plasma Aβ levels and Aβ42/40 ratios in diagnostic groups

The study cohort comprised 80 participants in total that were categorized into the diagnostic groups Aβ-positive (CSF Aβ42/40 ≤ 0.050, *n* = 38)) and Aβ-negative (CSF Aβ42/40 ratio > 0.050, *n* = 42). The measured Aβ40 levels in blood plasma did not differ statistically significantly between the two diagnostic groups. This held true for both direct plasma measurements without pre-treatment and measurements in IP-eluates (Table 2). In contrast, the Aβ42 levels and the Aβ42/40 ratios were statistically significantly lower in the Aβ-positive group in both plasma and IP-eluates. The magnitudes of the observed differences in medians between the diagnostic groups were 12.8% (Aβ42 in plasma), 16.2% (Aβ42 in IP-eluate), 16.1% (Aβ42/40 in plasma), and 15.5% (Aβ42/40 in IP-eluate).

**Table 2:** Aβ isoform levels and Aβ42/40 ratios measured on Elecsys without or with pre-analytical immunoprecipitation

	Aβ40 (plasma)	Aβ40 (IP-eluate)	Aβ42 (plasma)	Aβ42 (IP-eluate)	Aβ42/40 (plasma)	Aβ42/40 (IP-eluate)
*P*-value^1^	0.3906	0.5555	0.003	<0.0001	0.0003	<0.0001
Median of Aβ+	322.8	171.5	26.5	16.6	0.078	0.098
MAD of Aβ+	43.6	26.5	6.6	2.8	0.017	0.006
Median of Aβ−	326.7	174.3	30.4	19.8	0.093	0.116
MAD of Aβ−	45.3	27.1	6.4	4.2	0.013	0.010

^1^Mann-Whitney-test *p*-values for the comparison between the groups Aβ-positive (Aβ+) and Aβ-negative (Aβ−)

*Aβ* amyloid-β, *Aβ+* Aβ-positive: CSF Aβ42/40 ≤ 0.050, *Aβ−* Aβ-negative: CSF Aβ42/40 > 0.050, *MAD* median absolute deviation with scaling factor 1.4286

### Diagnostic performance of Elecsys measurements with and without pre-analytical IP for the discrimination between subjects with and without CSF biomarker evidence of brain amyloid

**Fig. 3:**
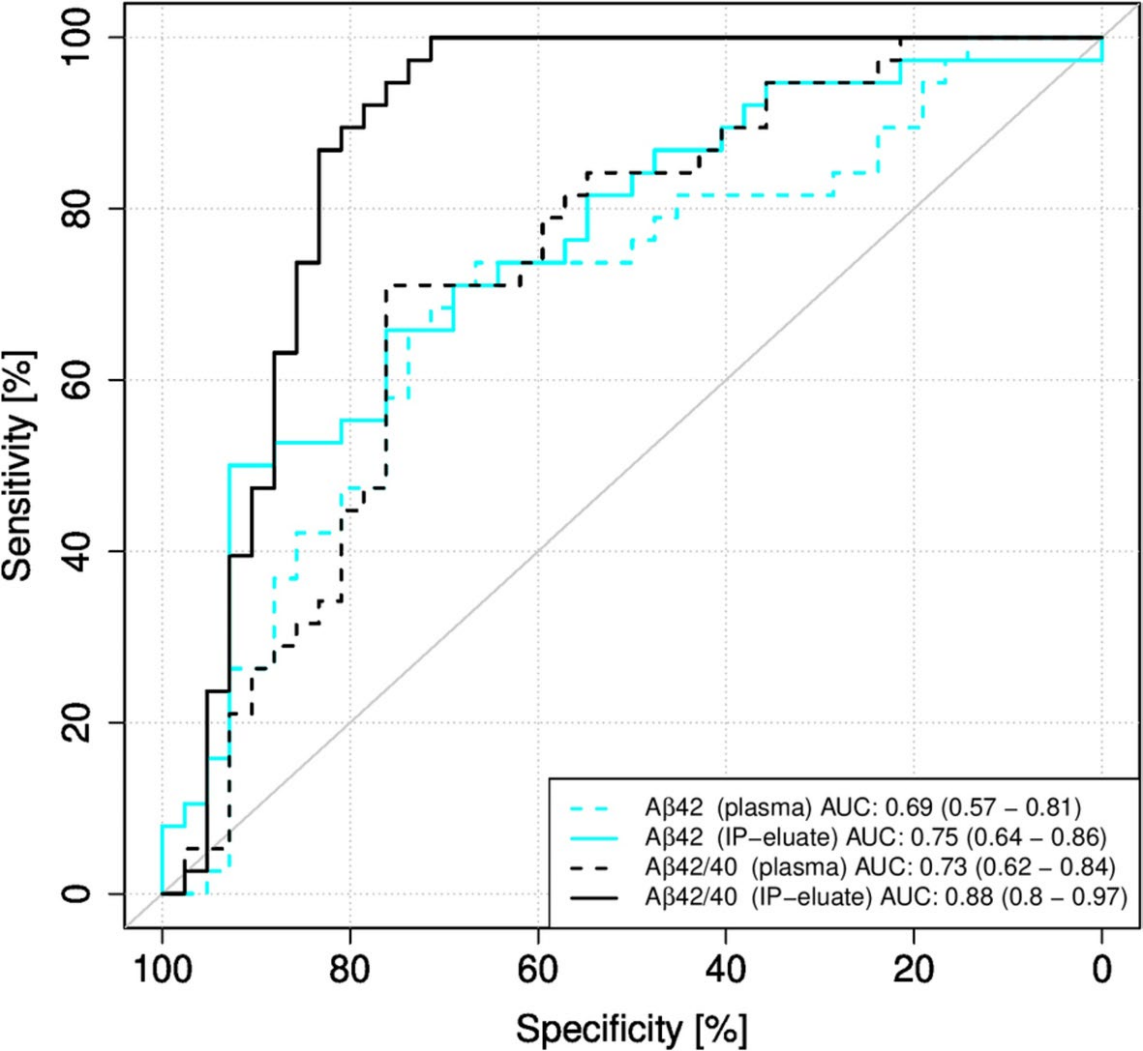
Receiver operating characteristic (ROC) curves for Elecsys measurements without or with pre-analytical immunoprecipitation (IP). ROC curves for the classification of the study participants into the diagnostic groups Aβ-positive (Aβ+) and Aβ-negative (Aβ−) were calculated for Elecsys measurements in plasma or IP-eluates. 95% confidence intervals were calculated using the DeLong approach and are indicated in brackets. Aβ, amyloid-β; Aβ+, Aβ-positive: CSF Aβ42/40 ≤ 0.050; Aβ−, Aβ-negative: CSF Aβ42/40 > 0.050; IP, immunoprecipitation

The performance of Aβ42 and the Aβ42/40 ratio in plasma determined on Elecsys with or without pre-analytical IP for the classification into the groups Aβ-positive and Aβ-negative was evaluated by ROC analysis (Fig. 3). Aβ40 was not included in this analysis, since there was no significant difference between the groups (Table 2). Pre-analytical magnetic bead Aβ IP increased the AUC for plasma Aβ42 from 0.69 to 0.75 (DeLong test *p*-value = 0.2184) and for the Aβ42/40 ratio from 0.73 to 0.88 (DeLong test *p*-value = 0.01547).

### Comparison of Aβ isoform measurements by two different immunoassay platforms

The data presented so far have provided preliminary evidence that pre-analytical magnetic bead IP can improve the diagnostic performance of automated plasma Aβ42/40 prototype assays on the Elecsys platform. Several Aβ immunoassays are available, and in previous studies, we employed a multiplex immunoassay kit with manual pipetting and plate washing for Aβ-isoform quantification in magnetic bead IP-eluates [21, 22]. For comparison with the automated Elecsys Aβ assays with pre-analytical sample workup reported above, we additionally analyzed corresponding Aβ IP-eluates (after 6-fold dilution) with the MSD Aβ panel 1 (6E10) multiplex assay kit. The technical within-plate (intra-assay) and between-plate (inter-assay) coefficients of variation (CVs) of MSD-measurements of diluted IP-eluates were calculated using two different control IP-eluate pools measured in 6 replicates on each of the 3 assay plates used. The averaged intra-assay CV was 6.1% for Aβ40 and 5.3% for Aβ42. The averaged between-plate CV was 3.2% for Aβ40 and 1.5% for Aβ42.

**Fig. 4:**
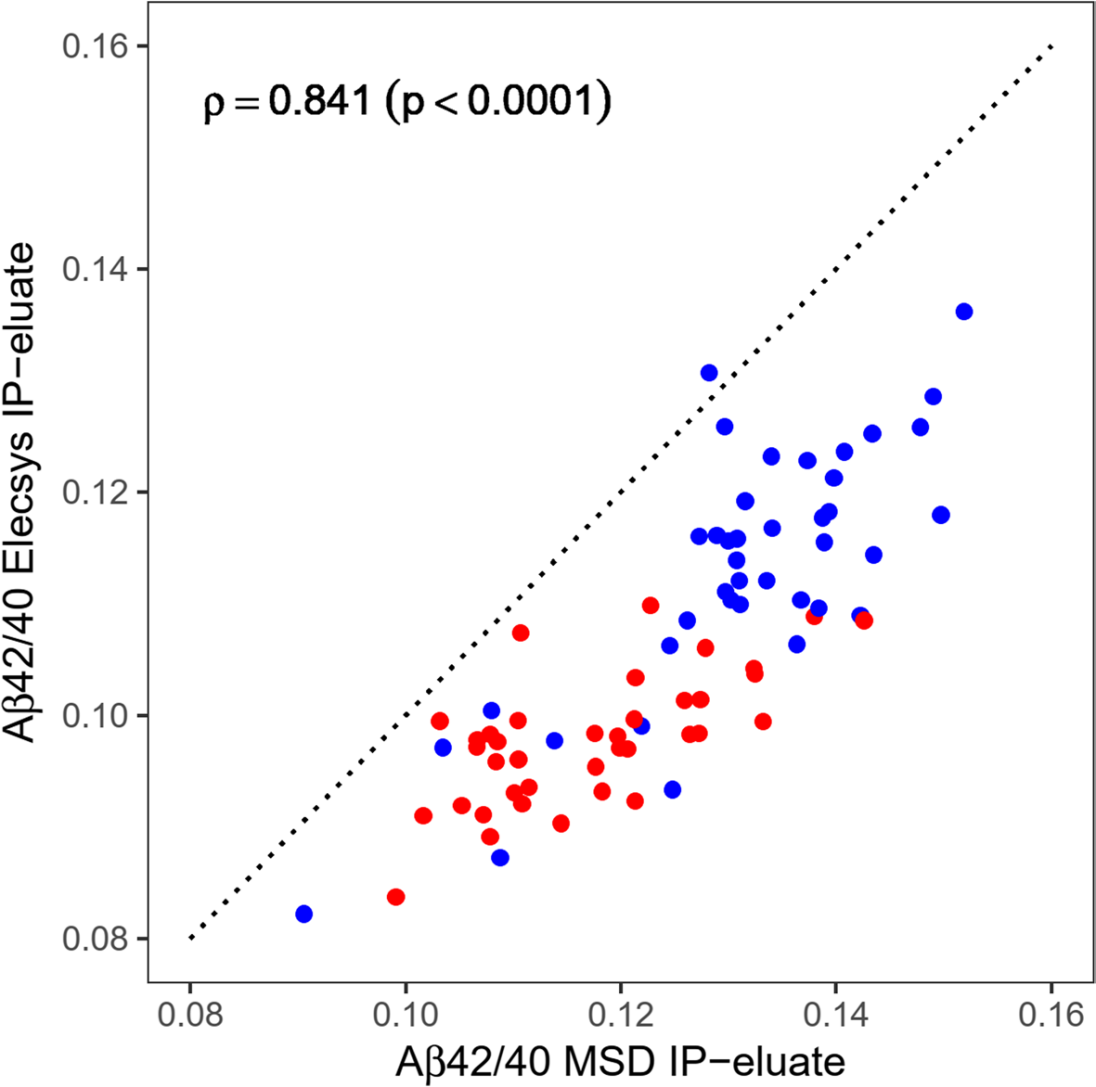
Correlation of plasma Aβ42/40 measurements after pre-analytical Aβ immunoprecipitation on two different immunoassay platforms. Following magnetic bead immunoprecipitation (IP) from EDTA blood plasma, Aβ42/40 was quantified in diluted IP-eluates with Elecsys (5-fold dilution) and MSD (6-fold dilution). Data points are colored by diagnostic group (red = Aβ+, blue = Aβ−). The diagonal dashed line corresponds to the identity line. Aβ, amyloid-β; Aβ+, Aβ-positive: CSF Aβ42/40 ≤ 0.050; Aβ−, Aβ-negative: CSF Aβ42/40 > 0.050; MSD, Meso Scale Discovery V-PLEX Aβ panel 1 (6E10) assay kit

The Aβ measurements on the two different immunoassay platforms correlated statistically significantly with Spearman correlation coefficients of *ρ* = 0.855 (Aβ40), *ρ* = 0.879 (Aβ42), and *ρ* = 0.841 (Aβ42/40 ratio) (Fig. 4).

The measured concentrations of Aβ40 and Aβ42 in 6-fold diluted IP-eluates according to the MSD multiplex assay were substantially lower than those of Aβ40 and Aβ42 in 5-fold diluted IP-eluates measured on the Elecsys platform. The median percent difference between MSD vs. Elecsys was − 46% (min–max: − 58 to − 32%) for Aβ40 and − 36% (min–max: − 51 to − 20%) for Aβ42.

**Fig. 5:**
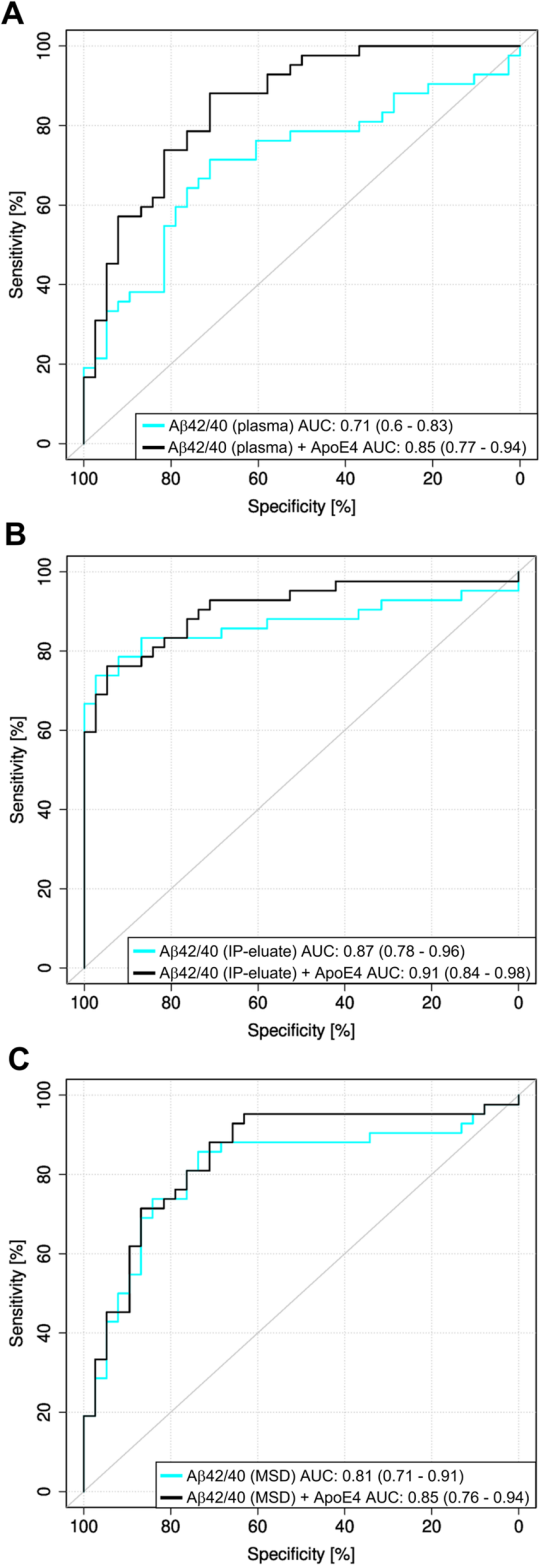
ROC curves for Aβ− vs. Aβ+ by logistic regression with single and bivariate models. Logistic regression receiver operating characteristic (ROC) analysis with leave-10-out cross-validation for single marker (blue lines) and bivariate models (including the ApoE4 allele frequency, black lines) for the Aβ42/Aβ40 ratio measured **A** on Elecsys in plasma without pre-analytical workup, **B** on Elecsys with pre-analytical Aβ immunoprecipitation (IP-eluate), and **C** on Meso Scale Discovery (MSD) V-PLEX Aβ panel 1 (6E10) assay with pre-analytical Aβ IP (IP-eluate). Aβ, amyloid-β; Aβ+, Aβ-positive: CSF Aβ42/40 ≤ 0.050; Aβ−, Aβ-negative: CSF Aβ42/40 > 0.050

ROC curves for Aβ42/40 for the classification of Aβ-negative vs. Aβ-positive with single and bivariate models were calculated by logistic regression for (i) Elecsys measurements in plasma, (ii) Elecsys measurements in IP-eluates, and (iii) MSD measurements in IP-eluates (Fig. 5). A statistically significant increase in the AUC by including ApoE4 allele frequency in the model was observed for the Elecsys assay without pre-analytical workup (*p* = 0.04) but not for Elecsys and MSD measurements in IP-eluates (*p* = 0.26 and *p* = 0.25, respectively).

Taking into consideration recent recommendations of the International Working Group on the clinical diagnosis of AD [23], additional exploratory post hoc ROC analyses using biomarker-supported clinical diagnosis as a second state variable were performed. Pre-analytical IP increased the ROC AUC for Elecsys Aβ42/40 from 0.77 (plasma) to 0.92 (IP-eluates) (DeLong test *p*-value = 0.01576) (additional Fig. 1). Comparisons of AUCs from logistic regression ROC analyses for the state variable CSF Aβ42/40 vs. biomarker-supported clinical diagnosis did not reveal statistically significant differences (additional Table 1).

### Correlations between CSF Aβ values and plasma Aβ

**Fig. 6:**
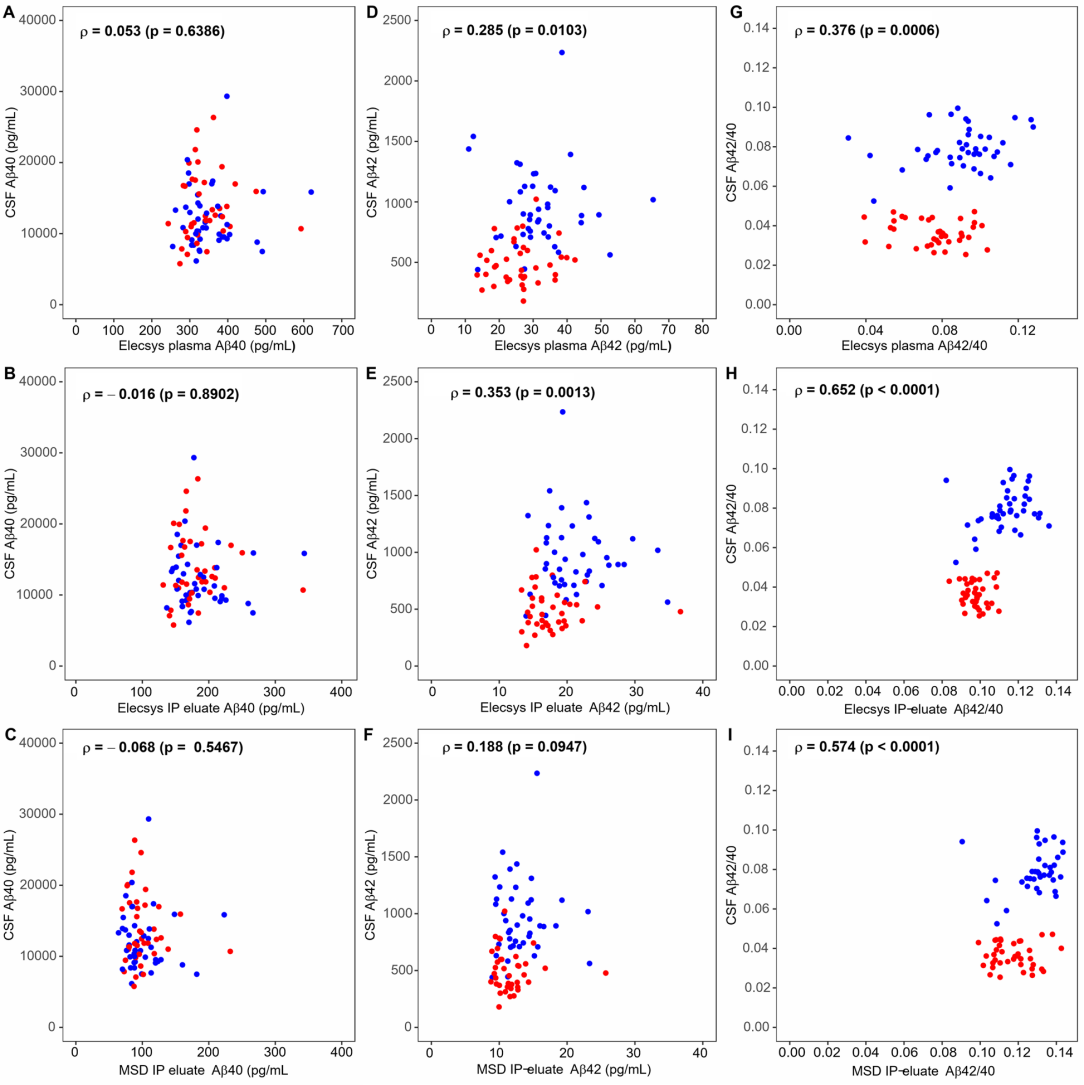
Correlations between CSF Aβ values and plasma Aβ measurements by three different assays. The cerebrospinal fluid (CSF) concentrations of Aβ40 (**A**, **B**, **C**), Aβ42 (**D**, **E**, **F**) and the CSF Aβ42/Aβ40 ratio (**G**, **H**, **I**) are plotted against the corresponding plasma values measured with Elecsys in plasma (**A**, **D**, **G**), Elecsys in IP-eluates (**B**, **E**, **H**) and MSD in IP-eluates (**C**, **F**, **I**). Spearman correlation coefficients and *p*-values are indicated. Data points are colored by diagnostic group (red = Aβ+, blue = Aβ−). Aβ, amyloid-β; Aβ+, Aβ-positive: CSF Aβ42/40 ≤ 0.050; Aβ−, Aβ-negative: CSF Aβ42/40 > 0.050; IP, immunoprecipitation; MSD, Meso Scale Discovery V-PLEX Aβ panel 1 (6E10) assay kit

Comparisons of Aβ40, Aβ42, and the Aβ42/40 ratio in CSF with corresponding plasma values measured with (i) Elecsys in plasma, (ii) Elecsys in IP-eluates, and (iii) MSD in IP-eluates are summarized in Fig. 6. CSF Aβ42/40 was statistically significantly correlated with Elecsys IP-eluate Aβ42/40 (*ρ* = 0.652, *p* < 0.0001), MSD IP-eluate Aβ42/Aβ40 (*ρ* = 0.574, *p* < 0.0001), and Elecsys plasma Aβ42/Aβ40 (*ρ* = 0.376, *p* = 0.0006). Furthermore, *p*-values smaller than 0.05 were observed for the correlations of CSF Aβ42 with Elecsys IP-eluate Aβ42 (*ρ* = 0.353, *p* = 0.0013) and Elecsys plasma Aβ42 (*ρ* = 0.285, *p* = 0.0103).

## Discussion

In this exploratory study, we examined the impact of semi-automated pre-analytical Aβ immunoprecipitation on the diagnostic performance of fully automated Elecsys prototype immunoassays for measuring the Aβ42/40 ratio in blood plasma. The pre-analytical sample workup resulted in a remarkable improvement in the accuracy of plasma Aβ42/40 in identifying subjects with low CSF Aβ42/40: in ROC analysis, the AUC increased from 0.73 (without pre-analytical sample workup) to 0.88 (after pre-analytical Aβ IP, *p* = 0.01547). This finding may be explained by amelioration of matrix interferences caused by high concentrations of other blood components in plasma [20, 24]. The current, partially automated IP protocol employs a monoclonal antibody against the Aβ amino-terminus and includes three different detergents during the antigen-antibody binding reaction. Thus, the IP procedure is expected to be highly efficient in removing other blood plasma components and thereby facilitating the subsequent immunological detection of Aβ.

The Aβ42/40 measurements in IP-eluates on Elecsys showed higher numerical AUC on ROC analysis than corresponding measurements in IP-eluates on MSD. Possible explanations may include the use of different antibody combinations and other assay reagents in the different assay kits and the fully automated workflow on the Elecsys platform vs. manual pipetting and washing steps in the MSD assay. However, in view of the limited sample size (*n* = 80) and the fact that only a single clinical cohort was studied, our observations have to be considered preliminary.

In a recent study comparing eight different plasma Aβ42/40 assays including Elecsys, Janelidze and colleagues found that two different IP-MS assays developed at Washington University and by Shimadzu, respectively, performed best in identifying subjects with abnormal CSF Aβ42/40. ROC analysis resulted in AUCs of 0.838–0.872 for the Washington University IP-MS assay (depending on the cohort or subcohort analyzed) and 0.825 for the Shimadzu IP-MS assay. The remaining six assays included four immunoassays, an antibody-free LC-MS assay, and another IP-MS method. Among the tested immunoassays, Elecsys reached the highest numerical AUCs in ROC analyses (0.773–0.795) [17]. In a different study, very similar AUCs of 0.77 for the plasma Aβ42/40 ratio and 0.80 when Aβ42 and Aβ40 were used as separate predictors in logistic regression analysis were observed with Elecsys assays [16]. For routine use in clinical settings, fully automated immunoassays, such as those performed, for example, on the Elecsys platform, are of particular interest because of the excellent technical performance that can be achieved [25]. We provide here evidence that the diagnostic accuracy of the Elecsys plasma Aβ42/40 prototype assays can be improved by pre-analytical Aβ IP. This finding may furthermore indicate that, possibly, other pre-analytical sample workup strategies or specific modifications of immunoassays could lead to similar improvements.

### Limitations

Limitations of this exploratory study include the use of a single, pre-selected clinical cohort and the relatively small size of the sample. Furthermore, the immunoassays (Elecsys vs. MSD) for measuring Aβ42/40 were performed at different laboratories, which may make a direct head-to-head comparison of the different technologies more difficult.

## Conclusions

In conclusion, our preliminary findings indicate that pre-analytical immunoprecipitation sample workup can improve the diagnostic performance of plasma amyloid assays. This observation may help to facilitate the further development of such blood-based assays for AD suitable for screening and routine use.

## Supplementary Information

**Additional file 1.** Second Endpoint: Biomarker-Supported Clinical Diagnosis.

## Data Availability

The datasets used and/or analyzed in the present study are available from the corresponding authors on reasonable request.

## Abbreviations

Aβ: Amyloid-β
AD: Alzheimer’s disease
AUC: Area under the curve
CSF: Cerebrospinal fluid
IP: Immunoprecipitation
IP-MS: Immunoprecipitation followed by mass spectrometry
MAD: Median absolute deviation scaled with factor 1.4826
MS: Mass spectrometry
PET: Positron emission tomography
pTau: Phospho-Tau
t-Tau: Total Tau
ROC analysis: Receiver operating characteristic analysis
