# Supplementary material for: Diagnostic performance of automated plasma amyloid-β assays combined with pre-analytical immunoprecipitation
Source: Alzheimers Res Ther. 2022 Sep 7;14:127. doi: 10.1186/s13195-022-01071-y (PMC9450259; doi:10.1186/s13195-022-01071-y)
Supplement: Supplementary file 1 — Additional file 1. Second Endpoint: Biomarker-Supported Clinical Diagnosis. [file 13195_2022_1071_MOESM1_ESM.docx]

**Additional File 1:**

**Second End Point: Biomarker-Supported Clinical Diagnosis**

**Classification of the study participants**

For additional post hoc analyses with a second endpoint, the study participants were classified into the diagnostic groups (i) probable or possible Alzheimer’s disease and (ii) improbable Alzheimer’s disease by biomarker supported clinical diagnosis. A multi-professional team consisting of three colleagues with expert experience related to the clinical differential diagnosis of neurodegenerative dementias (neuropsychologist, neurologist and psychiatrist), one physician from the UMG department of nuclear medicine with a clinical focus in Neuro-PET (FDG-PET, Amyloid-PET, DAT-Scan), and one neuroradiologist reviewed the clinical diagnostic data. The latter data comprised multi-domain psychometric testing (n=77), MRI or cCT (n=80), CSF biomarkers (n = 80) (Ratio Aβ42/Aβ40, phospho-Tau181, total-Tau), nuclear-medical examination (FDG-PET: n = 36; Florbetaben-Amyloid-PET: n = 16; DAT-Scan: n = 21) and clinical course. In three cases the dichotomized classification according to the CSF ratio Aβ42/Aβ40 had to be modified. In one case the CSF ratio Aβ42/Aβ40 was clearly decreased (0.028; cut-off ≤ 0.05), however, a normal amyloid-PET ruled out an Alzheimer disease associated dementia. Interestingly, the clinical course as well as the FDG-PET indicated that corticobasal degeneration was the more likely diagnosis. Therefore, this patient was re-classified as improbable AD. The second presented with a CSF ratio Aβ42/Aβ40 of 0.059 (cut-off ≤ 0.05) and accordingly was classified as control by the dichotomic CSF Aβ ratio dependent classification. Since his CSF Aβ42 concentration was decreased the expert laboratory for Clinical Neurochemistry (Prof. Piotr Lewczuk, University of Erlangen, Germany) had classified this patient as a possible case. Moreover, neuropsychological testing (amnestic dominant multidomain MCI), the short term clinical course (initially admitted because of severe depression, however, already in remission during neuropsychological testing), and pronounced atrophy of the medial temporal lobe (MTA score 3) also indicated possible Alzheimer´s disease. Accordingly, this patient was re-classified as possible AD. The third case presented with a CSF ratio Aβ42/Aβ40 of 0.052 (cut-off ≤ 0.05), again the CSF Aβ42 concentration was decreased. This case had been classified as probable AD by the laboratory for Clinical Neurochemistry, since his borderline normal CSF ratio Aβ42/Aβ40 was within the range of the technical inaccuracy of the assay applied for measurement. A pathological Amyloid-PET, the clinical course and the neuropsychological testing supported the diagnosis of probable Alzheimer´s disease. Accordingly, the patient was re-classified from Aβ-negative to probable AD. The latter clinical cases demonstrate that a synoptic multiparametric clinico-pathological classification improves the validity of a biomarker supported patient classification algorithm, as recently advocated by the International Working Group for Clinical Diagnosis of AD (Dubois *et al.* 2021). Principally, we suggest to apply both patient classification strategies in parallel since thus a high reliability and multi-site comparability can be combined with improved validity of the diagnostic algorithm. The latter will become even more important when blood based dementia diagnostics enters clinical routine and cut-off values have to be derived.


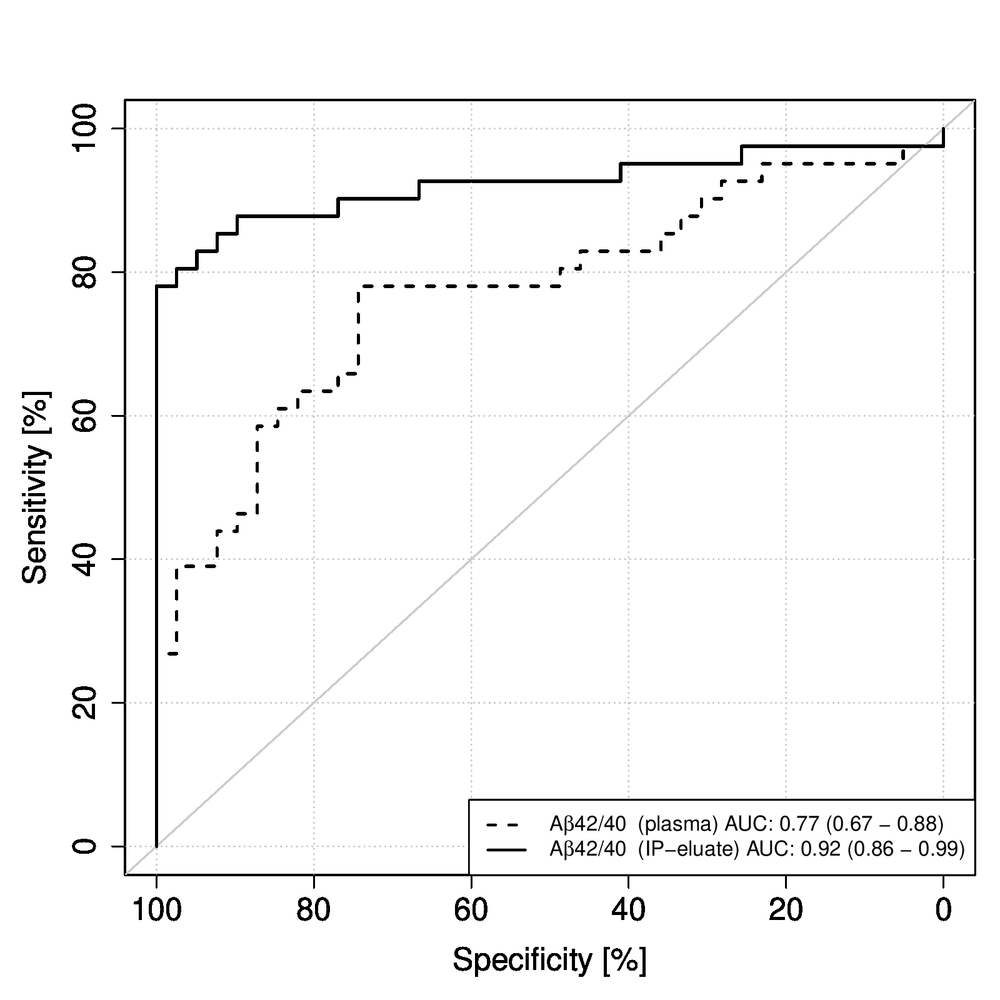


**Additional Figure 1:** Comparison of Receiver Operating Characteristics (ROC) curves for the state variable biomarker supported clinical diagnosis for Aβ42/40 determined by Elecsys measurements without (plasma) or with pre-analytical immunoprecipitation (IP-eluate). The increase in the AUC from 0.77 (Elecsys plasma) to 0.92 (Elecsys IP-eluate) reached statistical significance (p = 0.01576, DeLong test). Within the context of a blood screen test the pronounced increase of diagnostic sensitivity is of specific relevance.

**Additional Table 1.** Univariate and bivariate logistic regression ROC analysis for low cerebrospinal fluid (CSF) Aβ42/Aβ40 vs. biomarker supported clinical diagnosis (second end point).

|  | | **Univariate model AUC (95% CI)** | | | | | |
| --- | --- | --- | --- | --- | --- | --- | --- |
| **Aβ42/Aβ40 assay** | | **CSF Aβ42/Aβ40 ^1^** | | | **Biomarker supported clinical diagnosis ^2^** | **P-value ^3^** | |
| Elecsys plasma | | 0.73 (0.62–0.84) | | | 0.77 (0.67–0.88) | 0.59 | |
| Elecsys IP-eluate | | 0.88 (0.8–0.97) | | | 0.92 (0.86–0.99) | 0.47 | |
| MSD IP-eluate | | 0.83 (0.74–0.93) | | | 0.85 (0.76–0.94) | 0.73 | |
|  | | **Bivariate model including ApoE genotype AUC (95% CI)** | | | | | |
|  | | **CSF Aβ42/Aβ40 ^1^** | | | **Biomarker supported clinical diagnosis ^2^** | **P-value ^3^** | |
| Elecsys plasma | | 0.85 (0.77–0.94) | | | 0.84 (0.75–0.93) | 0.83 | |
| Elecsys IP-eluate | | 0.91 (0.84–0.98) | | | 0.93 (0.87–0.99) | 0.70 | |
| MSD IP-eluate | | 0.85 (0.76–0.94) | | | 0.85 (0.76–0.94) | 0.97 | |
|  |  | |  |  | | |  |

^1^ CSF Aβ42/Aβ40 was dichotomized according to the clinical cutoff ≤0.05.

^2^ The subjects were classified into the diagnostic groups (i) probable or possible Alzheimer’s disease and (ii) improbable Alzheimer’s disease by biomarker supported clinical diagnosis (see above).

^3^ DeLong test p-value for CSF Aβ42/Aβ40 vs. biomarker supported clinical diagnosis used as the state variable in ROC analysis.

**Reference:**

Dubois, B., Villain, N., Frisoni, G. B. et al. (2021) Clinical diagnosis of Alzheimer's disease: recommendations of the International Working Group. *The Lancet. Neurology,* **20,** 484-496.
